# LatinX harm reduction capital, medication for opioid use disorder, and nonfatal overdose: A structural equation model analysis among people who use drugs in Massachusetts

**DOI:** 10.1016/j.drugalcdep.2024.111293

**Published:** 2024-04-15

**Authors:** Shikhar Shrestha, Thomas J. Stopka, Jaclyn M.W. Hughto, Patricia Case, Wilson R. Palacios, Brittni Reilly, Traci C. Green

**Affiliations:** a Department of Public Health and Community Medicine, Tufts University School of Medicine, Boston, MA, United States; b Department of Behavioral and Social Sciences, Brown University School of Public Health, Providence, RI, United States; c Department of Epidemiology, Brown University School of Public Health, Providence, RI, United States; d Center for Health Promotion and Health Equity, Brown University, Providence, RI, United States; e Bouvé College of Health Sciences, Northeastern University, Boston, MA, United States; f School of Criminology & Justice Studies, University of Massachusetts, Lowell, MA, United States; g Massachusetts Department of Public Health, Bureau of Substance Addiction Services, Boston, MA, United States; h Opioid Policy Research Collaborative, Heller School for Social Policy and Management, Brandeis University, Waltham, MA, United States; i Department of Emergency Medicine, The Warren Alpert Medical School of Brown University, Providence, RI, United States

**Keywords:** Opioids, Overdose, Medication for opioid use disorder, Harm reduction capital

## Abstract

**Background:**

We introduce the concept of harm reduction capital (HRCap) as the combination of knowledge, resources, and skills related to substance use risk reduction, which we hypothesize to predict MOUD use and opioid overdose. In this study, we explored the interrelationships between ethnicity, HRCap, nonfatal overdose, and MOUD use among PWUD.

**Methods:**

Between 2017 and 2019, people who currently or in the past used opioids and who lived in Massachusetts completed a one-time survey on substance use history, treatment experiences, and use of harm reduction services. We fit first-order measurement constructs for positive and negative HRCap (facilitators and barriers). We used generalized structural equation models to examine the inter-relationships of the latent constructs with LatinX self-identification, past year overdose, and current use of MOUD.

**Results:**

HRCap barriers were positively associated with past-year overdose (b=2.6, p<0.05), and LatinX self-identification was inversely associated with HRCap facilitators (b=−0.49, p<0.05). There was no association between overdose in the past year and the current use of MOUD. LatinX self-identification was positively associated with last year methadone treatment (b=0.89, p<0.05) but negatively associated with last year buprenorphine treatment (b=−0.68, p<0.07). Latinx PWUD reported lower positive HRCap than white non-LatinX PWUD and had differential utilization of MOUD.

**Conclusion:**

Our findings indicate that a recent overdose was not associated with the current use of MOUD, highlighting a severe gap in treatment utilization among individuals at the highest risk. The concept of HRCap and its use in the model highlight substance use treatment differences, opportunities for intervention, and empowerment.

## 1. Introduction

In the US, drug overdose deaths have exceeded 500,000 between the years 2000 and 2020, with a majority of drug overdose-related deaths caused by opioids (Centers For Disease Control and Prevention, 2021). In recent years, the rate of opioid-related overdose deaths in LatinX individuals has significantly increased nationally as well as in high prevalence states like Massachusetts (Cano, 2020; Hoopsick et al., 2021; Stopka et al., 2023). These increases could result from racial/ethnic disparities in the use of medication for opioid use disorders (MOUD), the social stigma associated with substance use, a history of racial trauma and incarceration, and limited representation/peers in harm reduction, treatment, and recovery spaces among other factors (Andraka-Christou, 2021; Moran et al., 2022). Despite the life-saving nature of these treatments, both the duration and completion of MOUD treatment are reported to be lower among LatinX people (Mennis et al., 2019). Studies also report disparities in naloxone access (Kinnard et al., 2021) and racial/ethnic discrimination in healthcare settings, which can shape health outcomes in people who self-identify as LatinX (Pro and Zaller, 2020). Limited access to harm reduction service programs, limited support for safe drug use practice, stigma, and criminal justice system involvement present obstacles to harm reduction services and place people who use drugs (PWUD) at a higher risk of opioid overdose, particularly given widespread exposure to fentanyl in local drug supplies (Montaque et al., 2022; Palamar et al., 2022; Lofaro and Miller, 2021).

Harm reduction resources, including naloxone, sterile syringes, fentanyl test strips, overdose prevention sites, and education on safe injection practices, are vital to reducing the risk of opioid overdose along with low-barrier access to MOUD (Hawk et al., 2015; Clark et al., 2014; Peiper et al., 2019). However, such resources are predicated on a supportive social environment (Rhodes, 2009). For example, naloxone requires someone close by who knows how to administer it in the case of an overdose. The use of sterile syringes, without syringe sharing, also requires that all people within networks of PWUD use sterile syringes and avoid syringe sharing. Drug use networks, peer relationships, the availability of sterile syringes, and perceptions of risk all shape syringe-sharing practices (Rich et al., 2022; Dasgupta et al., 2019; Park et al., 2019). The uptake of harm reduction practices, particularly in ethnic and racial communities experiencing discrimination, depends on trust in institutions and the quality of interactions with social services, healthcare, and the criminal justice system (Lopez et al., 2022). A broader perspective, which includes individual knowledge of substance use disorder, harm reduction education, support from a close network of family, peers, and other people who use drugs, and overall societal acceptance of harm reduction practices determines the success of harm reduction programs (Uzwiak et al., 2021; Walley et al., 2013).

**Fig. 1.**
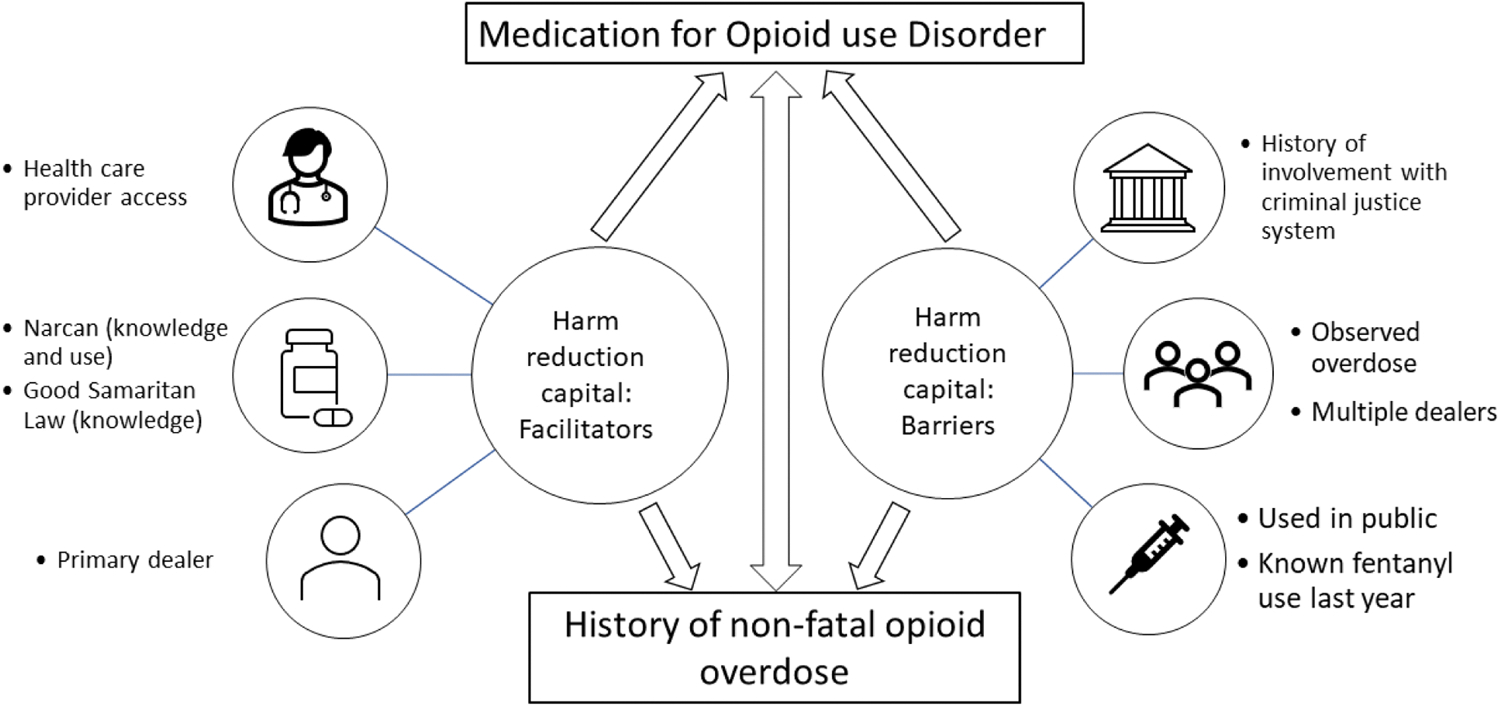
Conceptualization of Harm Reduction Captial and its association with medication for opioid use disorder and history of nonfatal opioid overdose.

There is a limited understanding of how opioid-related health outcomes are impacted by the relationship between individual knowledge of harm reduction services, access to such services, success in using such services, consistent harm reduction practices within peer networks, and local or state harm reduction policies. To understand this complex individual-environmental interaction, we derive the concept of harm reduction capital (HRCap) from the principles of (1) Social Capital (Putnam, 2001, 2015), which considers social networks as resources that enable the effective functioning of an individual within a society, (Puro and Kelly, 2022) and; (2) Recovery Capital, which includes individual and social resources that can be used to recover from substance use disorders (White, 2007; Granfield and Cloud, 1999). Strong familial and social bonds, the hallmarks of social capital, have been associated with better health (Ehsan et al., 2019). A decrease in social capital has been hypothesized to contribute to the emergence of the current opioid crisis (Sered, 2019). Improvement in Social Capital, particularly improvement in positive social networks developed within a recovery framework with high levels of trust, support, and social connectedness, is associated with lower rates of return to substance use and can act as a harm reduction measure (Panebianco et al., 2016; Cheung and Cheung, 2003; Weston et al., 2018). Using components of the Social and Recovery Capital model, we conceptualize HRCap (Fig. 1) as comprising two constructs: a) Harm reduction facilitators, including knowledge of harm reduction, use of harm reduction services, access to and use of healthcare providers, safe and reliable sources of illicit drugs, peers who utilize harm reduction practices, and networks of supportive friends and family; and b) Harm reduction barriers, which include: history of nonfatal overdose (NFOD), incarceration, unpredictable drug sourcing, lack of access to harm reduction services, living in areas with “not in my backyard” (NIMBY)-ism (public opposition towards the development of services and structures that have an overall positive impact but are deemed to be undesirable in their community), (Hunter, 2022) and punitive approaches to substance use (Bowen and Irish, 2019).

Our objective in this study was to examine the interrelationships between ethnicity, HRCap, history of nonfatal opioid-involved overdose, and MOUD use among people with a history of opioid and other drug use in Massachusetts. We hypothesize that HRCap facilitators would be positively associated with MOUD treatment whereas HRCap facilitators would be negatively associated with MOUD treatment. Our overall rationale is that HRCap facilitators as a set of skills would enable PWUD to seek MOUD treatment as they look to reduce their risk of adverse outcomes due to illicit drug use. Understanding variations in HRCap by ethnicity can inform public health action to address the disparities in health outcomes among PWUDs in the LatinX community.

## 2. Methods

### 2.1. Study population and recruitment

The Rapid Assessment of Consumer Knowledge (RACK) study conducted between August 2017 and November 2019 was a mixed methods assessment of PWUD in Massachusetts. To be eligible for the study, participants had to be 18 years of age or older, a Massachusetts resident, and report illicit substance use (excluding cannabis) in the last 30 days. Briefly, we sampled participants from municipalities in Massachusetts that showed an increase in fatal opioid overdose between 2016 and 2017. Using a combination of purposive and respondent-driven sampling, we recruited 469 PWUDs, including all racial/ethnic groups. All participants completed an interviewer-administered survey, which assessed sociodemographic variables, substance use history, opioid overdose experience, and knowledge of substance use and harm reduction. The Institutional Review Boards of the Boston University Medical Campus and Brandeis University approved the study. For this analysis, we selected a subset of study participants (n=429) who reported a current or past history of opioid use.

### 2.2. Study measures

#### 2.2.1. MOUD history

We asked study participants if they were taking either methadone or buprenorphine for opioid use disorders. We then created three binary indicator variables: a) for any MOUD treatment (either methadone or buprenorphine), b) treatment with methadone, and c) treatment with buprenorphine.

#### 2.2.2. Nonfatal opioid overdose history

We obtained data to assess self-reported lifetime and past-year experiences with NFOD by first asking study participants whether they had ever experienced a drug-related overdose. If participants answered in the affirmative, we asked about the month and year when they experienced their most recent overdose. We then calculated the time between the survey date and the date of the participant’s last overdose to determine if the NFOD had occurred during the past year.

#### 2.2.3. Harm reduction facilitators

We used a series of questions to assess harm reduction facilitators. The questions captured information on the respondent’s engagement in healthcare, availability and access to naloxone, drug purchase behaviors, and knowledge of Good Samaritan Laws. We created five harm reduction facilitator dummy variables: presence or use of a current doctor for regular health care, possession of a naloxone kit, perceived ease of access to naloxone, use of a primary dealer for drug purchase, and knowledge of Good Samaritan Laws.

#### 2.2.4. Harm reduction barriers

We defined harm reduction barriers as factors that added additional risk to drug use behaviors, and we used a series of questions to assess these barriers in the survey. The questions captured information on the participant’s involvement with the criminal justice system, overdose experiences, and drug purchase and use habits. We constructed five harm reduction barrier dummy variables: history of incarceration, history of observing someone having an opioid overdose, using multiple dealers for drug purchase, use of drugs in public in the past 30 days, and any self-reported and known use of fentanyl in the last year. We have listed the variables used for the estimation of the latent variables in the structural equation model in the appendix (Appendix Table 1).

#### 2.2.5. Sociodemographic variables

Ethnicity, gauged as whether the participant self-identified as LatinX (any race), was used as the primary independent variable in our model. We used gender, age, educational attainment, and current housing status as sociodemographic variables for the analysis based on the literature and our prior research. Based on the data distribution, we operationalized age into two categories: less than 30 years of age (young adults) vs. 30 years or older. We categorized educational attainment into two groups: less than high school and high-school graduate or higher, and housing status as binary: housed vs. unhoused. Data analysis Table 1

In the analyses, we used data from 429 individuals surveyed from 2017 to 2019. We calculated descriptive statistics for all study variables of interest. We used Chi-Square and Fisher’s Exact tests to examine global differences in sociodemographic variables between LatinX and non-LatinX groups. We fit first-order measurement constructs for HRCap as a) harm reduction facilitators and b) harm reduction barriers. Next, we used generalized structural equation models to examine the inter-relationship of the latent constructs with LatinX self-identification, last year overdose, and current use of MOUD. We used the GSEM function in STATA 17 (College Station, Texas) with a logit link function to account for the binary predictors and outcome variables in the model. We used the default estimator – Mean-variance adaptive quadrature (MVAGH). We present the fit statistics (Bayesian Information Coefficient: BIC) along with the standard coefficient and significance in the generalized structural equation model. The GSEM function in SATA does not calculate root mean square error of approximation (RMSEA) (Steiger, 1990) or confirmatory factor index CFI) (Bentler, 1990). Therefore, we conducted a secondary analysis using R and the Lavaan library and weighted least square mean and variance adjusted estimator (WLSMV) to fit the model. We report RMSEA from the secondary analysis.

## 3. Results

Approximately 75% of the study participants were above the age of 30 years, 60% were male, and 21% self-identified as LatinX. We found that 72% of the study participants had an education level of high school or more, and 66% were housed. Approximately 39% had experienced at least one nonfatal opioid overdose in the last year, 38% were currently receiving treatment for opioid use disorder, of which approximately 53% of participants were receiving buprenorphine treatment, and 47% were taking methadone.

LatinX individuals had lower educational attainment (58.6% had completed high school or had additional schooling) compared to non-LatinX individuals (77%, p<0.05). Additionally, a lower proportion of LatinX participants reported using drugs in public in the past 30 days (62.5% vs. 72.8%, p<0.05) and knowingly using fentanyl in the past year (77.7% vs. 86.2%, p<0.05) compared to non-LatinX participants. We also observed fewer harm reduction facilitators in LatinX participants: 68.8% of LatinX individuals had a primary dealer compared to approximately 90% of non-LatinX study participants (p<0.05); 79.2% of LatinX individuals reported easy access to naloxone compared to 88.3% in non-LatinX individuals (p<0.05); and only 67% had knowledge of Good Samaritan Laws compared to 85% in non-LatinX individuals (p<0.05). While there were no differences by ethnicity in past year NFOD or current MOUD treatment, a significantly lower proportion of LatinX individuals were currently on buprenorphine than non-LatinX participants (11.8% vs. 21.5%, p<0.05).

**Fig. 2.**
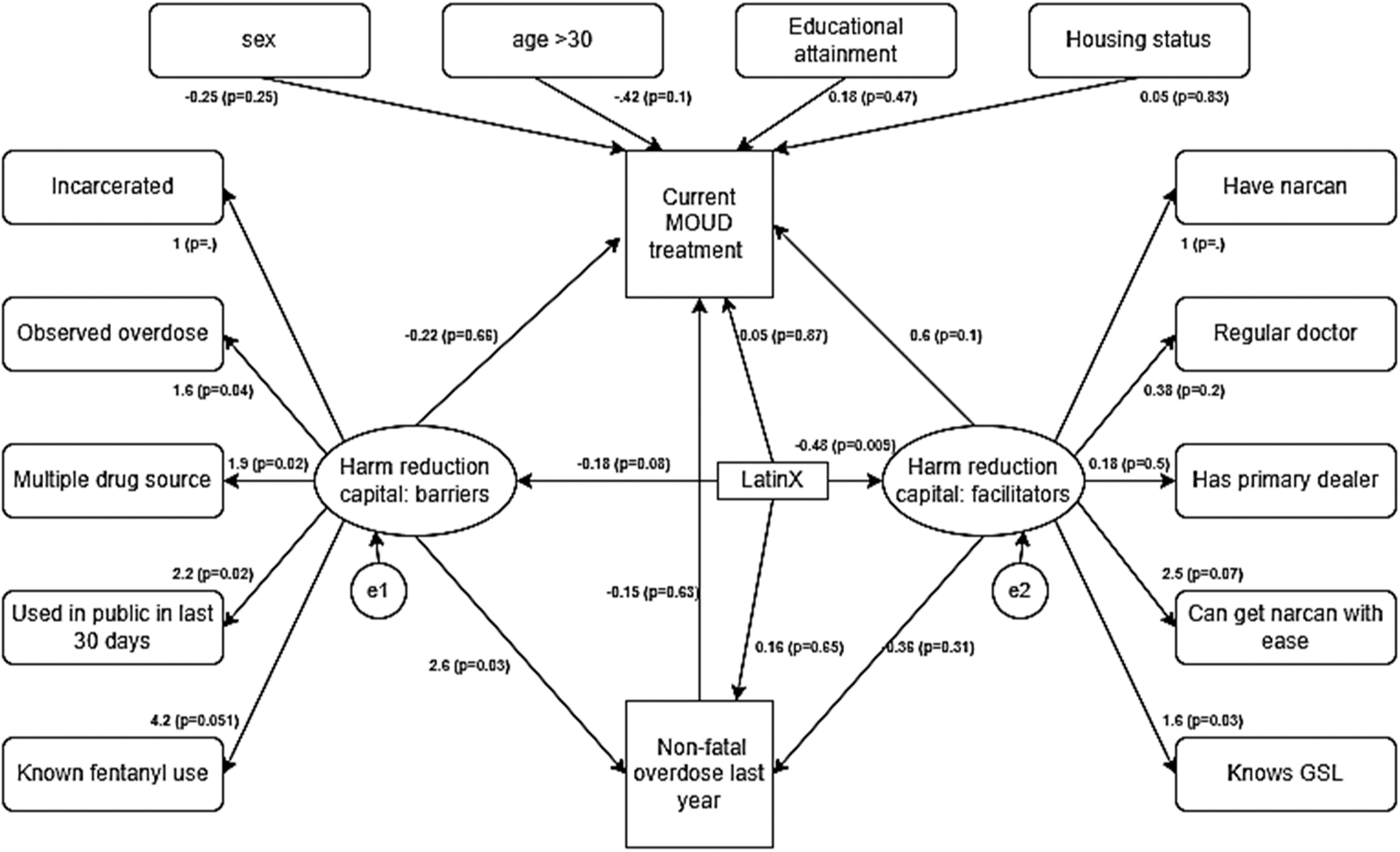
Structural equation model: Harm reduction capital and its association with current use of medication for opioid use disorder (either methadone or buprenorphine), history of nonfatal opioid overdose, and ethnicity. (n = 401, BIC: 5266.9, RMSEA (from secondary analysis): 0.059).

**Fig. 4.**
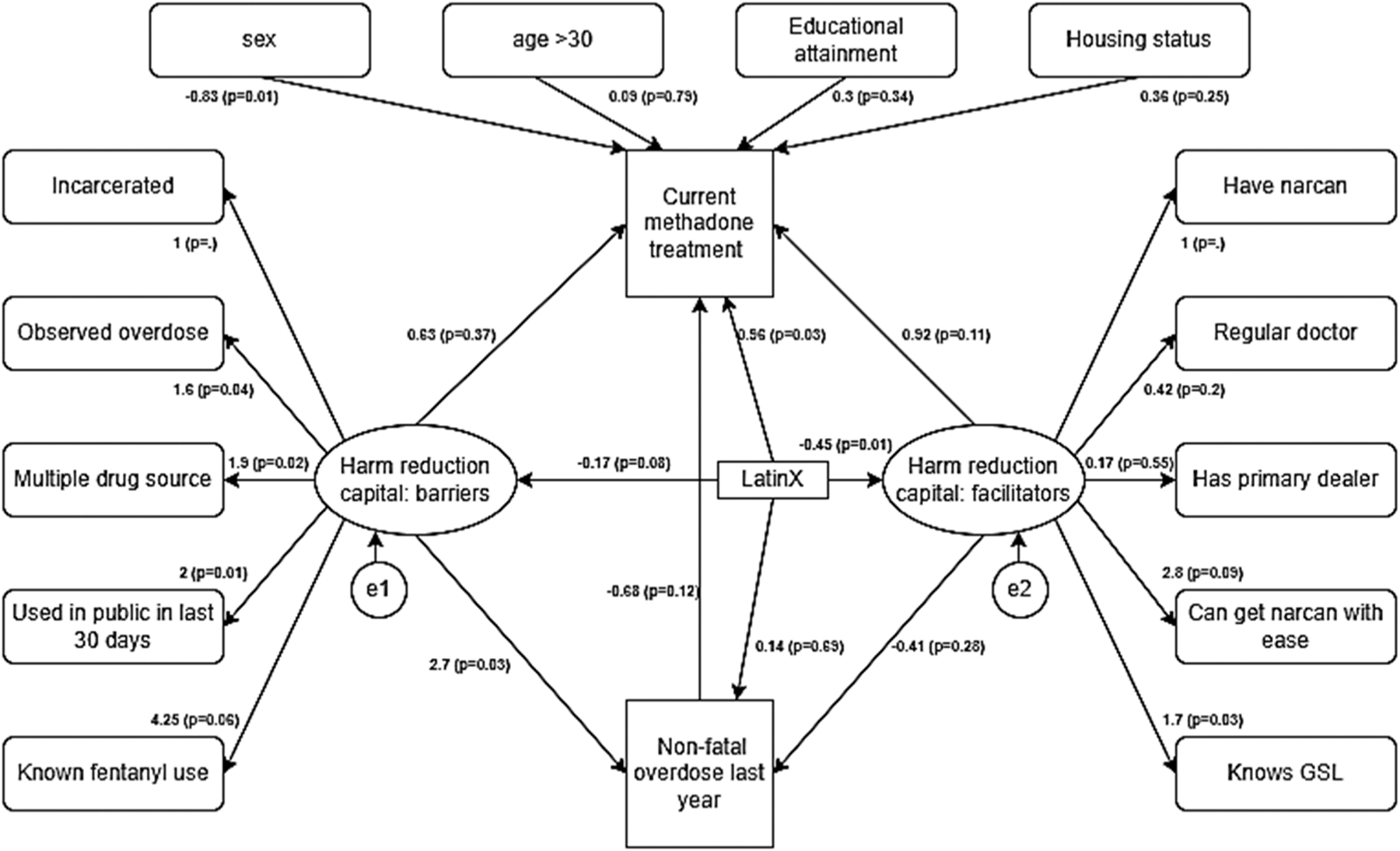
Structural equation model: Harm reduction capital and its association with current use of methadone, history of nonfatal opioid overdose, and ethnicity. (n = 402, BIC: 5225.8, RMSEA (from secondary analysis): 0.057).

Structural equation model estimates identified a statistically significant association between harm reduction constructs and outcome variables in the model. We observed that having more harm reduction barriers was associated with overdose in the past year (β = 2.6, p<0.05); harm reduction facilitators were not associated with past year overdose (Fig. 2. 3). The association between HRCap (both barriers and facilitators) and current MOUD treatment was not statistically significant. LatinX self-identification was associated with lower harm reduction facilitators (β = −0.49, p<0.05); the association between LatinX self-identification and harm reduction barrier was not statistically significant (β=−0.18, p=0.08). While having an overdose in the past year was not associated with current MOUD treatment, LatinX self-identification was positively associated with methadone treatment (β = 0.89, p<0.05, Fig. 4), but negatively associated with buprenorphine treatment (β = −0.68, p=0.07, Fig. 2).

## 4. Discussion

Effective harm reduction strategies are critical to preventing opioid overdose and minimizing the risk of transmission of infectious diseases associated with injection drug use. We present the concept of HRCap as a collection of individual measures, including knowledge, skills, self-efficacy, support systems, and peer networks that encourage and practice harm reduction and a collection of environmental practices and policies that promote harm reduction strategies. In our evaluation, we assessed HRCap and its association with NFOD experiences and utilization of MOUD in municipalities with higher rates of fatal opioid overdose in Massachusetts in LatinX vs. non-LatinX populations. We observed that more harm reduction barriers were associated with overdose in the last year. For LatinX individuals, we observed lower harm reduction facilitators and lower utilization of buprenorphine for OUD compared to non-LatinX individuals. We also found that a recent nonfatal overdose was not associated with current MOUD use. These findings highlight a significant gap in treatment utilization among individuals at the highest risk for opioid overdose, particularly LatinX individuals.

Lower dissemination or uptake of harm reduction services in communities of color has been documented in prior studies (Kinnard et al., 2021; Dayton et al., 2020), with additional difficulties in accessibility stemming from the COVID-19 pandemic (Rosales et al., 2022). A recent study in Massachusetts showed the lower distribution of naloxone from opioid education and naloxone distribution programs to racial/ethnic minorities compared to White populations (Nolen et al., 2022). Lower HRCap in LatinX individuals could indicate a lack of culturally-informed harm reduction services (Callejas et al., 2021; Urbanoski et al., 2020) and a lack of trust in institutions (Lopez et al., 2022). In addition, internalized stigma could lead to reduced engagement with programs that provide sterile syringes (Rivera et al., 2014). These findings present opportunities for intervention to promote culturally informed harm reduction practices and education among people at the highest risk.

Our study also found lower current utilization of MOUD among LatinX individuals, along with a lower proportion of LatinX individuals receiving buprenorphine compared to non-LatinX study participants. Our findings are similar to other reports that show lower uptake of buprenorphine in LatinX populations (Hadland et al., 2017; Hansen et al., 2013; Schiff et al., 2020). The differential uptake of methadone compared to buprenorphine in LatinX individuals indicates underlying disparities in treatment access. These disparities can stem from the limited availability of substance use treatment providers (Langabeer et al., 2020), particularly in less urban areas with a higher population of minorities (Hirchak et al., 2022). Historically, methadone clinics have been located in communities with predominantly Black or Hispanic residents and buprenorphine providers in white neighborhoods, therefore limiting access to buprenorphine treatment (Stein et al., 2018; Goedel et al., 2020). Furthermore, racial trauma, a history of criminalization, and a lack of trust in institutions could have also contributed to the disparity (Lopez et al., 2022). Also problematic is the observation that some studies indicate a negative attitude towards MOUD among minority communities (Husain et al., 2023; Zaller et al., 2009). Broader availability of various treatment options with low barriers to entry can help individuals initiate and maintain OUD treatment that best suits their needs (Lee et al., 2019).

Our study also finds that individuals who had a recent NFOD tended not to be on MOUD. While post-overdose outreach programs have shown to be protective against future overdoses (Xuan et al., 2023), our finding suggests a significant gap in treatment utilization in high-risk individuals. Multiple factors motivate treatment initiation after a nonfatal opioid overdose, such as availability and access to services, acceptability of services, patients’ past experience with OUD treatment programs, and their willingness to initiate treatment (Roy et al., 2021). Harm reduction barriers were also a strong indicator of last year’s nonfatal overdose, which highlights the effectiveness of harm reduction efforts in reducing opioid overdose. Furthermore, our study is likely to underestimate this association as individuals who had fatal overdoses are not included in the study—vulnerability among subpopulations with higher harm reduction barriers warrants further investigation.

Our study, however, did not find a strong association between HRCap and MOUD use in the study population. Possible explanations for lack of evidence for association include non-specific operationalized variables for the latent variable model, small sample size, and the overall study inclusion criteria. Since study participants were individuals who had used illicit substances in the past 30 days, it is likely that we captured a small fraction of people who were on MOUD and had not used illicit drugs such that the association between HRCap and MOUD use would be indeterminable.

There are several limitations of this study. Our findings primarily rely on self-reported data, which may be subject to recall bias and the under-reporting of substance use and opioid overdose events. Harm reduction behaviors have been shown to reduce the risk of opioid overdose, and since this cross-sectional assessment does not include data from decedents, our estimates of the impact of harm reduction practices could be lower than the true effect. Prospective studies on the use of harm reduction services and their impact on overdose events can lead to better estimates of their impact. In the era of complete contamination and adulteration of the illicit drug supply, there is a need for extensive prospective evaluation of social networks, drug use habits, and harm reduction behavior. The findings also have limited generalizability as the assessments were primarily done in municipalities that had higher rates of opioid overdoses but also, in recent years, were more supportive of harm reduction initiatives, as indicated by the presence of syringe service, overdose education, and naloxone distribution programs. Assessments of consumer knowledge in cities and towns with limited harm reduction and substance use treatment infrastructure could help alleviate these limitations. Furthermore, the smaller sample size of LatinX participants limited additional analysis.

In conclusion, findings from our study highlight the differences in HRCap by ethnicity of study participants living in communities with high rates of opioid overdose in Massachusetts. Differences in HRCap point to the need for additional resources, training, and interventions for vulnerable LatinX populations to reduce their risk of opioid overdose, utilization of harm reduction services, and initiation of MOUD. HRCap can be a useful and measurable concept that can differentiate disparities from equities.

## Figures and Tables

**Fig. 3.**
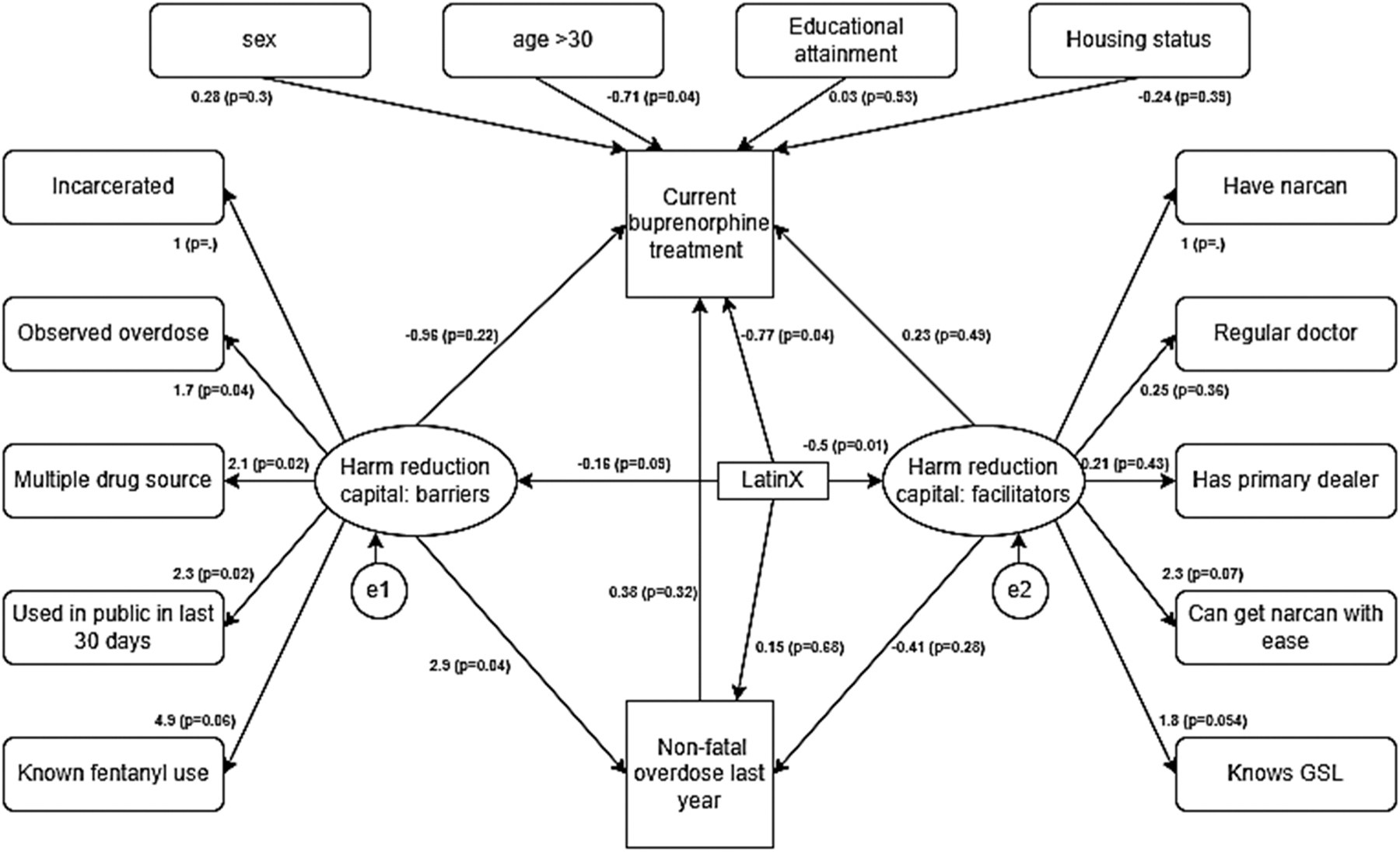
Structural equation model: Harm reduction capital and its association with current use of buprenorphine, history of nonfatal opioid overdose, and ethnicity. (n = 426, BIC: 5419.9, RMSEA (from secondary analysis): 0.06).

**Table 1:** Descriptive statistics by LatinX ethnicity, Massachusetts, 2018–2019 (n=429).

	Hispanic/LatinX				P-value
	Yes		No		

*Sociodemographic variables*					
**Sex**					0.008
Female	32	(29.1%)	137	(43.5%)	
Male	78	(70.9%)	178	(56.5%)	
**Age**					0.623
Over 30 years old	86	(76.8%)	236	(74.4%)	
30 years old or younger	26	(23.2%)	81	(25.6%)	
**Educational attainment**					<0.001
High school or more	65	(58.6%)	244	(77.0%)	
Less than high school	46	(41.4%)	73	(23.0%)	
**Housing status - current**					0.341
Unhoused	33	(29.5%)	109	(34.4%)	
Housed	79	(70.5%)	208	(65.6%)	
*Harm reduction barriers*					
**History of incarceration**					0.49
Yes	67	(60.4%)	203	(64.0%)	
No	44	(39.6%)	114	(36.0%)	
**Witnessed an overdose - lifetime**					0.153
Yes	98	(87.5%)	289	(92.0%)	
No	14	(12.5%)	25	(8.0%)	
**Multiple sources for drug purchase**					0.713
Yes	94	(85.5%)	262	(84.0%)	
No	16	(14.5%)	50	(16.0%)	
**Used drugs in public - past 30 days**					0.041
Yes	70	(62.5%)	230	(72.8%)	
No	42	(37.5%)	86	(27.2%)	
**Used fentanyl - past year**					0.04
Yes	80	(77.7%)	269	(86.2%)	
No	23	(22.3%)	43	(13.8%)	
*Harm reduction facilitators*					
**Has a regular healthcare provider**					0.318
Yes	70	(63.1%)	215	(68.3%)	
No	41	(36.9%)	100	(31.7%)	
**Currently has a naloxone kit**					0.66
Yes	72	(64.3%)	211	(66.6%)	
No	40	(35.7%)	106	(33.4%)	
**Has a primary dealer**					0.03
Yes	64	(68.8%)	206	(79.8%)	
No	29	(31.2%)	52	(20.2%)	
**Easy access to naloxone**					0.02
Yes	80	(79.2%)	263	(88.3%)	
No	21	(20.8%)	35	(11.7%)	
**Knowledge of Good Samaritan Law**					<0.001
Yes	62	(67.4%)	218	(85.2%)	
No	30	(32.6%)	38	(14.8%)	
*Outcome variables*					
**Nonfatal opioid overdose last year**					0.675
Yes	42	(37.5%)	126	(39.7%)	
No	70	(62.5%)	191	(60.3%)	
**MOUD treatment - current**					0.368
Yes	34	(34.3%)	119	(39.4%)	
No	65	(65.7%)	183	(60.6%)	
**Buprenorphine treatment - current**					0.026
Yes	13	(11.8%)	68	(21.5%)	
No	97	(88.2%)	248	(78.5%)	
**Methadone treatment - current**					0.353
Yes	21	(21.0%)	51	(16.9%)	
No	79	(79.0%)	251	(83.1%)	

Note. MOUD = Medications for Opioid Use Disorder

## Appendix

**Table 1:** Questions from the survey for Variables used in the structural equation model.

Variables used in SEM model	Questions from the survey

Incarceration	Have you ever been incarcerated?
Witnessing overdose	How many overdoses have you ever witnessed? Please provide a number from 0 to 100.
Multiple Dealers	About how many different people do you buy or get drugs from (dealers, friends, relatives, etc.)?
Drug use in public	In the last 30 days, have you used drugs in a public place (for example, park, sidewalk, public or restaurant bathroom, at the beach, stairwell)?
Known use of fentanyl	In the past year, have you used fentanyl or drugs you thought or knew to have fentanyl in them?
Have Narcan	Do you currently have a naloxone/Narcan kit with you or in the place where you use drugs?
Primary Dealer	Is there one person who you consider your primary dealer (you will always go to them first to buy)?
Knowledge of Good Samaritan Laws	Have you heard of the Massachusetts’s Good Samaritan Law?
Ease of accessing Narcan	Around here, how easy would you say it is to get naloxone to take home with you?
Regular doctor	Do you have a doctor that you regularly see for health care?
